# Ordered Self-Assembly Mechanism of a Spherical Oncoprotein Oligomer Triggered by Zinc Removal and Stabilized by an Intrinsically Disordered Domain

**DOI:** 10.1371/journal.pone.0036457

**Published:** 2012-05-09

**Authors:** Clara Smal, Leonardo G. Alonso, Diana E. Wetzler, Angeles Heer, Gonzalo de Prat Gay

**Affiliations:** 1 Fundación Instituto Leloir and Instituto de Investigaciones Bioquímicas-Conicet, Buenos Aires, Argentina; 2 XBio Inc., Buenos Aires, Argentina; 3 Departamento de Química Biológica, Facultad de Ciencias Exactas y Naturales, Universidad de Buenos Aires, Ciudad Universitaria, Buenos Aires, Argentina; National Institute for Agricultural Research, France

## Abstract

**Background:**

Self-assembly is a common theme in proteins of unrelated sequences or functions. The human papillomavirus E7 oncoprotein is an extended dimer with an intrinsically disordered domain, that can form large spherical oligomers. These are the major species in the cytosol of HPV transformed and cancerous cells. E7 binds to a large number of targets, some of which lead to cell transformation. Thus, the assembly process not only is of biological relevance, but represents a model system to investigate a widely distributed mechanism.

**Methodology/Principal Findings:**

Using various techniques, we monitored changes in secondary, tertiary and quaternary structure in a time course manner. By applying a robust kinetic model developed by Zlotnik, we determined the slow formation of a monomeric “Z-nucleus” after zinc removal, followed by an elongation phase consisting of sequential second-order events whereby one monomer is added at a time. This elongation process takes place at a strikingly slow overall average rate of one monomer added every 28 seconds at 20 µM protein concentration, strongly suggesting either a rearrangement of the growing complex after binding of each monomer or the existence of a “conformation editing” mechanism through which the monomer binds and releases until the appropriate conformation is adopted. The oligomerization determinant lies within its small 5 kDa C-terminal globular domain and, remarkably, the E7 N-terminal intrinsically disordered domain stabilizes the oligomer, preventing an insoluble amyloid route.

**Conclusion:**

We described a controlled ordered mechanism with features in common with soluble amyloid precursors, chaperones, and other spherical oligomers, thus sharing determining factors for symmetry, size and shape. In addition, such a controlled and discrete polymerization reaction provides a valuable tool for nanotechnological applications. Finally, its increased immunogenicity related to its supramolecular structure is the basis for the development of a promising therapeutic vaccine candidate for treating HPV cancerous lesions.

## Introduction

It is well established that proteins have no unique conformation; in fact, protein conformation in solution differs depending on the chemical and physical parameters under which they are studied. The different conformations acquired may include the native protein ensemble, soluble oligomers of different morphology, and insoluble amyloid fibrils, among other structures.

Amyloid like structures have been observed *in vitro* from disease-associated and disease-unrelated proteins and peptides, and despite having different folding topologies and characteristics, they show common properties, such as the formation of spherical soluble oligomeric precursors [1], [2]. However, the formation of spherical soluble oligomers in biological systems is not limited to the formation of amyloid fibril precursors but also to other relevant systems like chaperone proteins [3], [4], viral origin binding proteins [5], [6], spherical nanoclusters, “Blackberry” type supramolecular structures or self assembly macroions [7]. Besides morphological and structural similarities between these different non-related oligomers, they can be grouped based on their kinetic assembly mechanism. In addition, the formation of viral capsids follows a similar kinetic mechanism [8]. Kinetic mechanisms of protein self-assembly of closed spherical oligomers is poorly understood due to experimental difficulties on assaying an assembling system that involves different (and at the same time similar, i.e., the same subunit) species and time scales.

The human papillomavirus is a small DNA tumor virus, the causative agent for uterine cervix cancer and other types of cancers of high impact on health. They have two main oncoproteins, E6 and E7, which are responsible for tumorigenic progression, with counterparts in other small DNA tumor viruses that operate by forcing cells into S-phase in order to use the cell machinery for replication of their viral genomes [9], [10]. E7 is the major transforming protein in HPV and is under the repressive control of the E2 master regulator [11]. The E2 open reading frame is disrupted upon integration of the viral genome to the host chromosome, and thus, in the absence of the repressor, the E7 oncoprotein expression becomes deregulated, promoting transformation [12]. We and others have shown that there is a direct interaction between E2 and E7 [13], [14], and careful biochemical investigation led us to propose a finely tuned mechanism for regulating the relative protein levels of E2 and E7 and effects on the balance between repression and transformation, based on oligomerization and aggregation of the complexes formed [14].

In this work we investigate the particular case of the oligomerization mechanism of E7 protein from human papillomavirus as a model for protein self-assembly [15], [16]. HPV16 E7 is a 98-amino acid protein bearing two domains, the N-terminal, E7N, and the C-Terminal, E7C, of 40 and 58 aminoacids, respectively. E7 was initially described as an extended dimer, which can be described at least in part as an intrinsically disordered protein (IDP) [17], [18]. The intrinsically disordered property was found to map to the E7N region, which was defined as a *bona fide* highly conserved domain, despite lacking canonical secondary or tertiary structure. This, together with the absence of folding cooperativity, defines it as an intrinsically disordered domain (IDD) [19]. E7C is a dimeric folded domain that contains two highly conserved CXXC motifs which coordinate one mol of Zn per mol of protein [17], [20]. Structures of E7C from other related strains showed a well-structure domain with a non classical Zn finger-type arrangement [21], [22]. This metal was shown to be fundamental for maintaining the E7C fold and as a prerequisite for dimerization [22].

In a previous work, we demonstrated that E7 can self-assemble *in vitro* into spherical oligomers (E7SOs) when the protein Zn is removed by a chelating agent [15]. E7SOs are highly stable, they show homogeneous size and morphology, and bind to dyes like Congo Red and thioflavin T, reflecting the presence of a repetitive β-sheet structure in the non-fibrillar self-assembly of E7SOs. Interestingly, it was shown that E7SOs, and not the E7 dimer, can bind and prevent aggregation of non-viral proteins normally used as standard chaperone substrates. The topological arrangement of these oligomers indicate that the E7C forms the oligomerization core, while the E7N IDP domain faces the solvent [16].

E7 has been reported to have nuclear and cytoplamatic localization [23], [24], [25], [26]. We have shown that the oligomeric forms of E7 are in fact present in model HPV-transformed cell lines and cancerous tissue in cell, with cytoplasmic localization, while the E7 dimer-monomer shows nuclear localization [27]. In addition, we found that the cytosolic oligomer represents the majority of the E7 protein in these cells. Although the precise nature of these cellular oligomers cannot be determined, the conformation repetitive β-sheet structure was confirmed by co-localization of thioflavin-S staining and E7 in inmunofluorescence experiments [27]. Altogether, these results provide a strong biological relevance for the investigation of this assembly mechanism in connection with the transformation properties of this prototypic viral oncoprotein.

The E7SOs display a non-reversible and complete assembly process with an optimal time-scale to be probed in detail with different spectroscopic and biophysical techniques. In this work, we present a kinetic dissection of the E7SOs assembly mechanism. We show that the reaction can be analyzed by the application of the kinetic model proposed by *Zlotnick et al* [28]. The reaction is triggered by zinc removal, progresses through a lag phase into an elongation phase, to yield the final stable soluble complex. We elucidated the intermediate size and the different structural events involved in E7 assembly process: E7C is the oligomerization domain and the E7N intrinsically disordered domain provides solubility and prevents progression into an insoluble fibrillar route.

Due to the dynamic nature of the process, the ability to control it can be particularly in pathological cases in order to target the assembly as a therapeutic strategy [29], [30], [31], [32] and in nanotechnology applications in order to design suitable disposals of desired size [33], [34].

## Results

### Time Course Events in E7SOs Assembly

**Figure 1:**
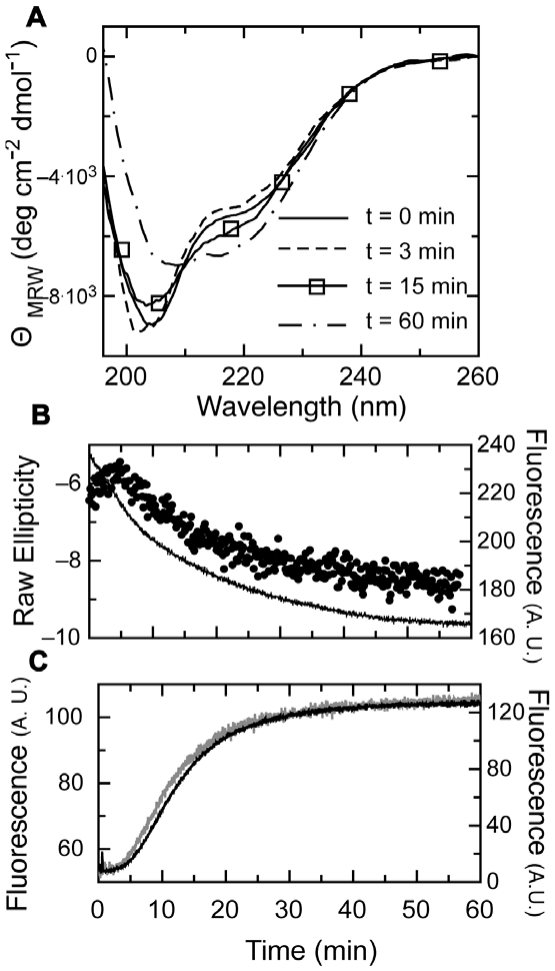
Assembly Kinetics of E7SOs followed by different spectroscopy probes. (A) CD spectra obtained at different times during E7SOs assembly. CD spectrum of E7_2_ before addition of EDTA (full line) and CD spectra of E7 at different times after EDTA addition, at 3 minutes (dashed line), 15 minutes (full line with rectangles) and at 60 minutes (dashed and dotted line). (B) Kinetics of E7SOs assembly monitored by circular dichroism and tyrosine fluorescence. Far-UV CD at 216 nm (black circles) and tyrosine fluorescence (full line); both experiments were carried out at the same protein concentration and triggered by the addition of 1.0 mM EDTA. (C) Kinetics of E7SOs assembly followed by Thioflavin T fluorescence (black line) and ANS fluorescence (grey line); both experiments were carried out at the same protein concentration and triggered by the addition of 1.0 mM EDTA right before beginning the measurement.

As an initial approach to investigate the assembly mechanism of the E7SOs we analyzed the time course events using different spectroscopic probes. As we previously showed, the formation of the oligomers is accompanied by a change in secondary structure, as judged by the far UV CD spectra [15], [17]. The reaction is triggered by the addition of EDTA to the folded dimeric form of E7 (E7_2_), and spectra at different times show two points of maximum signal change, one around 202 nm and the other at 216 nm (Figure 1A). Although only two species (folded dimer and oligomer product) are observed at the equilibrium, the spectrum at 3 minutes showed a decrease in the 220 nm region. In fact, when we monitored the change in ellipticity with time at 202 nm and 216 nm, a biphasic behavior was observed at the two wavelengths (Figure 1B, 216 nm is shown). A first rearrangement is over by 300 seconds, followed by a slower second transition with a half-life of <11 minutes (Fig. 1B).

The change in tertiary structure was followed by monitoring the intrinsic fluorescence change corresponding to tyrosine residues. There is a slow decrease in fluorescence with an apparent t_1/2_ <11 minutes, reaching a steady state at 60 minutes (Figure 1B). Taking advantage of the amyloid-like properties of E7SOs we have previously described [15], we aimed at monitoring the formation of repetitive β-sheet upon assembly of the oligomer, as a probe for quaternary structure. We analyzed the kinetics by adding thioflavin T (ThT) and measuring the change in fluorescence with time. For this experiment, we determined that the binding of the dye takes place within the dead time of the experiment (15 seconds, not shown), much faster than that of the oligomerization itself, a noticeably slow process. The process shows a lag phase of <250 seconds, not observed in the tertiary structure monitored by tyrosine fluorescence (Figure 1B), but coincident with the first secondary structure rearrangement (Figure 1C). This lag phase is followed by a slow increase in ThT binding with a half-life approximately of 10 minutes at this protein concentration, which is in overall agreement with the tertiary structure rearrangement observed by tyrosine fluorescence change. In any case, the lag phase is a strong indicator of an intermediate species being accumulated.

As an additional probe, we tested the evolution of the binding of 8-anilino, 1-naphtalene sulfonate (ANS) with time. ANS binds to hydrophobic environments or cavities, when these are accessible to the solvent, constituting an alternative probe for evaluating the formation of tertiary and quaternary structure [35]. Figure 1C shows that the ANS fluorescence change is superimposable to the ThT binding trace, strongly suggesting that they are monitoring similar events, i.e., concomitant tertiary and quaternary structure formation, and coincident with the slow CD rearrangement. It should be stressed that no detectable fast phase is observed within the experimental dead time, in any of the probes used.

**Figure 2:**
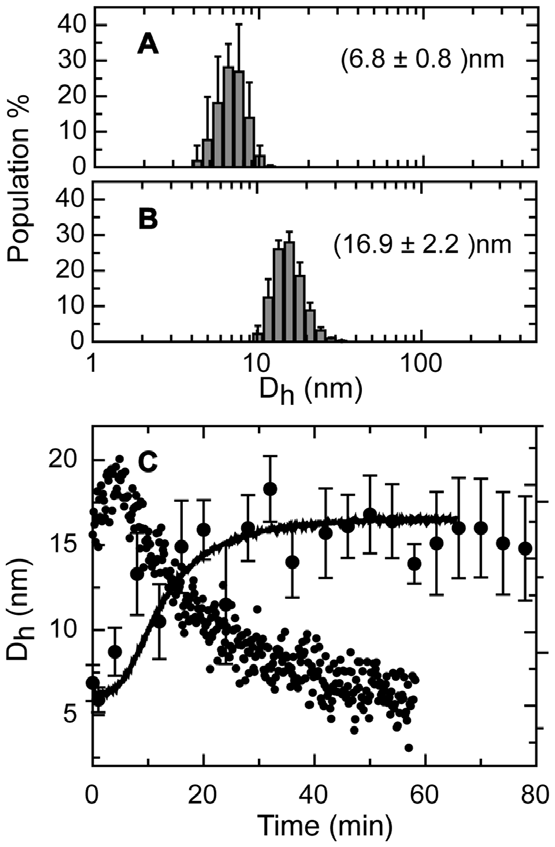
Size distribution measured by DLS. (A) Size distribution profile of E7 dimer before addition EDTA. (B) Size distribution profile of E7SOs. (C) Kinetics of E7SOs formation followed by DLS (black circles with error bars), where the initial time correspond to E7 dimer before the addition of EDTA, kinetics followed by Th T fluorescence (black line) and far-UV CD at 216 nm (black circles). The experiments were carried out at the same protein concentration and triggered by the addition of 1.0 mM EDTA.

The extremely slow nature of the process and the presence of multiple phases, allow for the determination of the size increase as the reaction proceeds. For this purpose, we measured the time course assembly of the E7SOs by dynamic light scattering (DLS). The hydrodynamic diameter (D_h_) of E7 species was measured before the addition of EDTA and after the stationary state was reached (Figure 2A, 2B). The species at time zero that corresponds to E7 dimer yields a D_h_ = 6.8±0.8 nm, larger than the expected size for a globular protein of 196 amino acids (4.4 nm [36]). This observation is in excellent agreement with previous results where we determined an extended conformation of E7_2_ in solution, originated from its intrinsically disordered nature [17]. The hydrodynamic diameter of E7SOs was measured in the plateau of the reaction, yielding a D_h,E7SOs_ of 16.9±2.2 nm (Figure 2B).

If we superimpose the DLS data with CD data, we can conclude that changes in tertiary and quaternary structure indeed take place in parallel (Figure 2C). However, since ThT fluorescence (similar to ANS) yields a much better signal-to-noise ratio, and is superimposable with CD (Figure 2C), we use ThT for subsequent experiments which require fiting the data to a mathematical model.

To further investigate the size of the species formed in the lag phase, we measured the diameter at 1 and 4 minutes, with D_h_ values of 5.9±0.7 nm and 8.7±1.4 nm, respectively. The experimental value D_h,1min_ is in accordance with the predicted value of a denatured chain of the same length than the E7 monomer (6 nm [36]). However, as D_h,1min_ and D_h,4min_ are similar to the diameter found for the E7 dimer (6.8±0.8 nm) within experimental error, we cannot distinguish between monomer or dimer at this “pre-oligomerization” phase. Nevertheless, these species are clearly smaller than the endpoint oligomers (16.9±2.2 nm), indicating that the slow phase corresponds to the oligomerization event.

### Zinc Removal as the Trigger for E7SOs Assembly

Coordination of Zn is essential for folding of the C-terminal domain of E7, and a prerequisite for dimerization [22]. Since oligomerization is triggered by the removal of Zn upon addition of a chelating agent, we wanted to asses how a compound with different metal affinity might affect the reaction and its phases. We used N,N,N′,N′-tetrakis(2-pyridyl-methyl)ethylenediamine (TPEN) a chelator with a two order of magnitude higher affinity constant for Zinc with respect to EDTA [37]. TPEN has specificity for heavy metals like Zn (Ka 10^16^ M^–1^) but low affinity for Ca^+2^ and Mg^+2^ (Ka 10^4^ M^–1^ and 10^2^ M^–1^, respectively), providing a higher selectivity [38].

**Figure 3:**
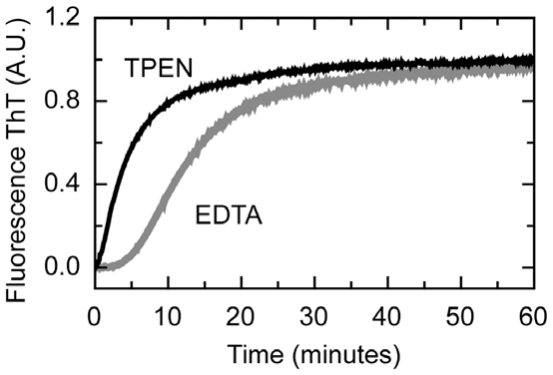
Assembly of E7SOs triggered by different Zn chelators. Kinetics assays were followed by Th T fluorescence. The reaction was started with different chelating metal, 1.0 mM EDTA (grey full line) and 1.0 mM TPEN (black full line) right before beginning the measurement. The protein concentration was 15 µM in both experiments.

We monitored the oligomerization kinetics followed by ThT fluorescence for both chelators (Figure 3). CD spectra and DLS confirmed that the final oligomers obtained after the addition of both chelators are similar (not shown). Figure 3 shows that TPEN noticeably shortens the lag phase, although the slow oligomerization phase appears unaffected (t_1/2(EDTA)_<8 min y t_1/2(TPEN)_ <6 min), but clearly the largest effect is observed in the lag phase. The disappearance of the lag phase is likely to arise from the higher affinity of TPEN for zinc, which accelerates the formation of the metal-free intermediate, without affecting the polymerization phase. This strongly suggests that the rate-limiting step is the sequential break of the individual zinc-thiolate bonds from the protein, ultimately leading to complete metal removal.

**Figure 4:**
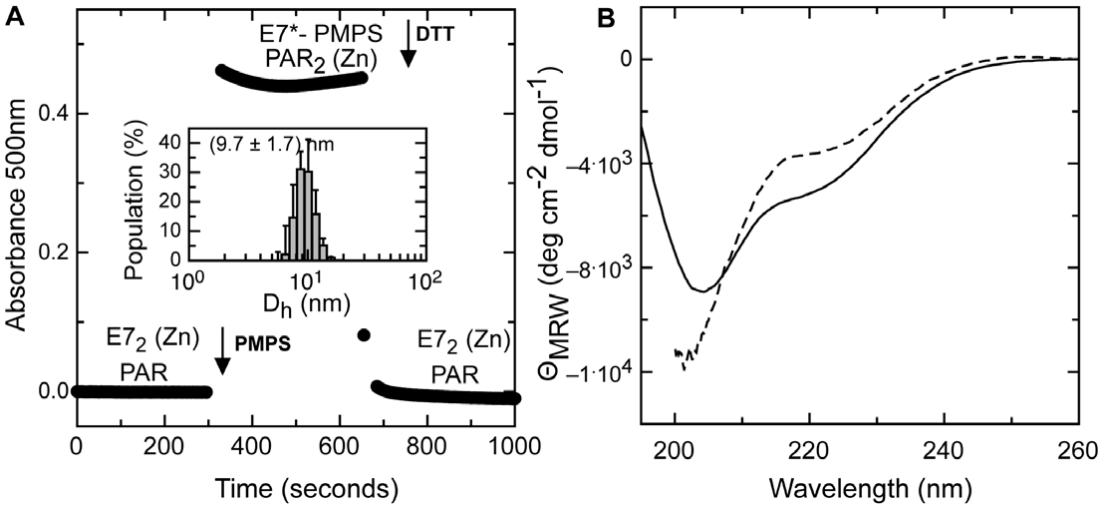
Evaluation of cysteine blocking and Zn release effect on E7 conformation and oligomerization state. (A) Analysis of zinc release from the high affinity coordination center of E7 followed at 500 nm by the formation of Zn(PAR)_2_ complex (black circles). The addition of PMPS and DTT is indicated by arrows. Inset, size distribution profile of E7-PMPS measured by DLS. (B) CD spectrum of E7 (black line) and CD spectrum of E7 upon addition of PMPS (dashed line).

Next, we wanted to address the possibility of removing the zinc atom by blocking the cysteine groups instead of chelating the metal. For this, we made use of *p*-hydroxymercuriphenylsulfonate (PMPS) an organomercurial compound which reacts instantly with cysteine residues, displacing the metal from the high-affinity Zn coordinating center. The release of the zinc atom is reported spectroscopically by the formation of the complex of the metal with the metallochromic reporter e 4-(2-pyridylazo) resorcinol (PAR). When PMPS is added to a mixture of PAR with E7, the absorbance increase reports the stoichiometric formation of a Zn(PAR)_2_ complex, after the release of the metal from the protein coordination centre (Figure 4A). The PMPS modified apo-E7 is stable in solution but with a significant secondary structure change, namely, loss of alpha helical content as judged by FAR-UV CD spectrum (Figure 4B). This species does not form oligomers, even after prolonged incubation periods, and this can be confirmed by DLS which yields an hydrodynamic diameter of 9.7±1.7 nm (Figure 4A, inset DLS).

The addition of a reducing agent such as DTT should instantly break the PMPS-cysteine bonds, and the recapturing of the metal would indicate that the protein remains in a competent conformation for restoring the tetrahedral coordination of the zinc. This is the case for E7-PMPS, where addition of DTT instantly and stoichiometrically decreases the PAR-zinc absorbance back to the untreated baseline value (Figure 4A). An important implication of this result is that removal of zinc only may not be enough to trigger polymerization; a conformational factor involving the cysteines is required for the formation of the oligomerization competent intermediate. Similar results were found when cysteines were modified with iodoacetamide (data not shown).

### Kinetic Model for the E7SOs Assembly Mechanism

The assembly of a spherical oligomer or a capsid can take place through multiple elementary reactions either combined or fragmented in intermediate species of different shapes and masses. Zlotnick *et al* developed simple and general models that can be applied to the formation of viral capsids or of any spherical polymer [28], [39]. These assembly processes can be described in terms of a cascade of low-order association reactions, which display a sigmoideal kinetic behavior with a lag phase that precedes the elongation phase to yield the final oligomer at the stationary state. Two models were proposed for interpreting the assembly mechanisms: the equilibrium assembly model (EQ) and the kinetically limited assembly (KL). The details of each model were explained in detail [28], but we briefly summarize the grounds for our choice of the model. We shall first clarify that the term “nucleation” used by Zlotnik is different from classical nucleation as observed in linear polymerization and typical amyloid routes [40], therefore, in order to avoid confusion, we name it “Z-nucleation”. The EQ model: i) does not require Z-nucleation, ii) requires low interaction energy among the monomers, iii) is susceptible to kinetic traps because of multiple parallel assembly initiation processes, and iv) the stability of the final assembly must be low. Z-nucleation is required in the KL model, and there is no restriction to the association energies or the stability of the final assembly. Z-nucleation refers to the formation of the minimum assembly competent unit, not to intermediate size oligomers, referred as “nucleus” in classical amyloid fiber models. More importantly, the KL model is robust to kinetic traps, which translates into a completed reaction and an homogeneous final assembly. Since these premises are satisfied in the reaction we describe, we apply the KL model for the analysis of our experimental data.

In the KL model, the Z-nucleation size and the elongation reaction order can be analyzed by spectroscopic probes [28]. We studied the E7SOs assembly monitoring the ThT fluorescence change that reports the oligomerization concomitant with the formation of repetitive β-sheet strands (see Figure 2C). We verified that the increase in ThT fluorescence is linearly dependent on protein concentration in the range used in these experiments (not shown).

**Figure 5:**
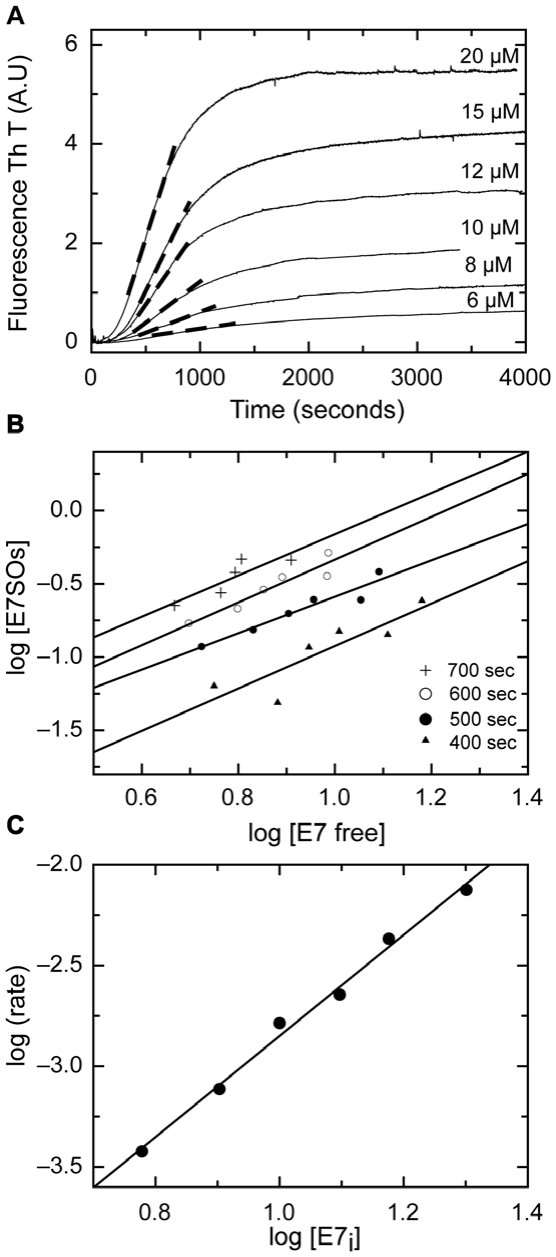
Kinetics of E7SOs assembly at different initial E7 concentration. (A) The assemblies of E7SOs at the indicated protein concentrations were monitored by ThT fluorescence. The rates of the elongation phase were estimated from the slope of dashed lines. (B) Determination of the nucleus size. Based on the KL model, the nucleus size *n* is calculated by averaging four slopes obtained from the log-log plots of the E7SOs versus E7 free molar concentration for different single time points as described in material and methods. According to this model, the observed nucleus size was 1.2±0.2. (C) Determination of the reaction order of elongation phase. The reaction order of E7SOs assembly was calculated of the power dependence of the rate of the elongation phase with the E7 initial concentration. The slope of the log-log plot 2.3±0.3 determines the elongation reaction as second order reaction.

The concentration dependence of the assembly process shows the concentration dependence of the rate and the extent of E7SOs formation (Figure 5A). In addition, two important parameters can be obtained from the time traces of oligomer formation at different protein concentrations based on the KL model. This model assumes that once formed, each z-nucleus should quickly form E7SOs and, therefore, the rate of the E7SOs formation is equal to the overall rate of z-nucleus production. The ratio [E7SOs]/[E7] is obtained at a given time within the linear elongation phase at each concentration. The z-nucleus size *n*, i.e., the number of component units can be determined from the following equation,
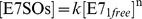
(1)


The double log plot allows for the determination of the z-nucleus size, in this case *n* = 1.2±0.2. This number is an average obtained from the plots at 4 different times (Figure 5B), indicating that the initial z-nucleus is monomer. As we had previously determined the “apo” nature of this species from the TPEN experiment (Figure 3), we can conclude that the z-nucleus is an apo-monomer. In support to this, the lag phase does not change significantly with concentration (Figure 5A) and is abolished by the stronger chelator TPEN (Figure 3). The weak dissociation constant of the E7 dimer (1 µM, [41]) implies that the dissociation rate will be much faster than the slow events related to the metal removal by EDTA, and the monomeric species (the starting point of the reaction) is instantly available (fast pre-equilibrium). Therefore, the apo-monomer acts as the building block for the assembly.

According to the model, the order of reaction of the elongation process was determined from the slope of the linear elongation phase, representing the rate of oligomerization, plotted against the initial concentration [E7]_i_ [28], [42]. This concentration dependence was measured in a log-log plot for six different concentrations; the slope was 2.3±0.3, indicating a second-order elongation reaction (Figure 5C). This result suggests a rapid sequential addition of single monomers as the pathway for E7SOs assembly proceeds. However, since i) the second-order addition is coupled to a slow conformational rearrangement, ii) the number of subunits was previously estimated to be 70 [15], iii) the overall process is completed in 2000 seconds at 20 µM, the overall average rate, calculated from the linear phase, is one monomer added every ∼28 seconds.

### The C-terminal Domain of E7 Drives the Oligomerization that Leads Fibril Formation in the Absence of the N-terminal Domain

E7 is a modular protein in all HPV types, where the C-terminal E7 domain (E7C) is the dimerization and Zn binding domain and the N-terminal IDD (Intrinsically Disordered Domain) domain displays different biologically relevant interaction sites. As an alternative way to probe the assembly mechanism and the regions involved, we decided to investigate the oligomerization of the isolated E7C by removing the N-terminal intrinsically disordered domain (IDD), by expressing the truncated form recombinantly.

**Figure 6:**
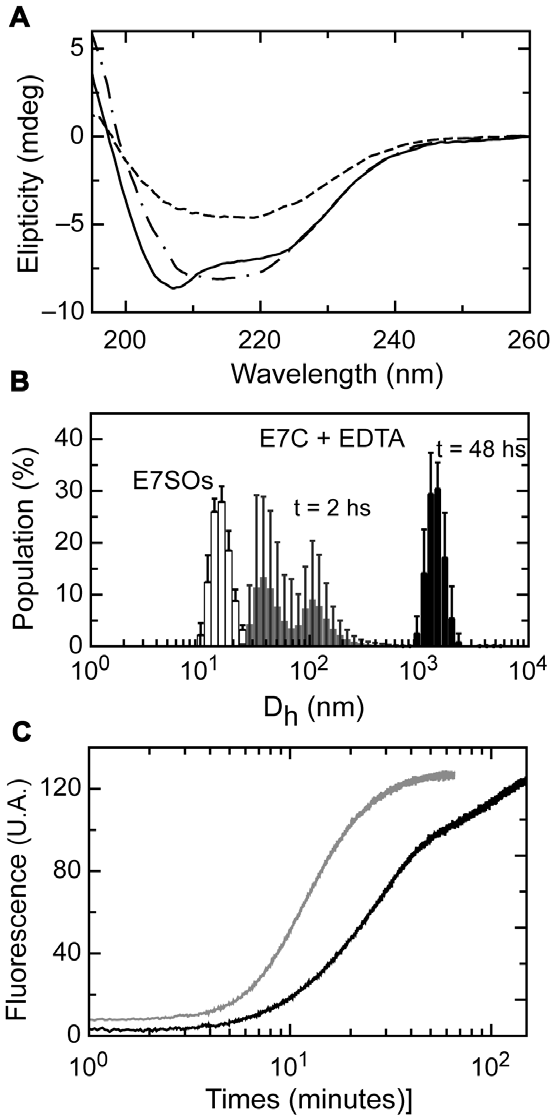
Oligomerization properties of E7C. (A) CD spectra of E7C (full line), E7C protein after 2 hours (broken and dotted line) and overnight incubation (broken line) with EDTA. (B) Size distribution measured by DLS. The distribution profiles correspond to: E7SOs (white bars), E7C incubated 2 hours with EDTA (grey bars) and E7C incubated 24 hours with EDTA (black bars). (C) Kinetic measurement of the oligomerization assembly after the addition of EDTA monitored by Thioflavin T fluorescence. E7C oligomerization kinetic (black full line) and E7SOs kinetic formation (grey full line). The time axis is in a log scale.

E7C is a globular dimeric domain with a CD spectrum typical of high α-helical content, with minima at 208 nm and 222 nm (Figure 6A) [21], [22]. The spectrum obtained after incubation with EDTA for approximately 2 hours indicates an increased proportion of β-sheet structure, with a broad minimum at around 216 nm. Further overnight incubation shows the loss of the signal, caused by insoluble aggregation. Similar to the full-length E7 protein, the reaction proceeds to an oligomer, but of larger size, more heterogeneous, and ultimately leading to large insoluble material of <2 nm under stirring conditions (Figure 6B, DLS).

The oligomeric product is also capable of binding of ThT and Congo Red (not shown), confirming the β-sheet repetitive or amyloid-like conformation. The kinetic reaction was followed by fluorescence of ThT (Figure 6C) with a lag phase (<270 seconds) similar to that observed for full-length E7, but with a slower elongation phase that did not reach the plateau after <150 minutes, in agreement with the overlapping subsequent slow formation of an insoluble aggregate (Figure 6B).

**Figure 7:**
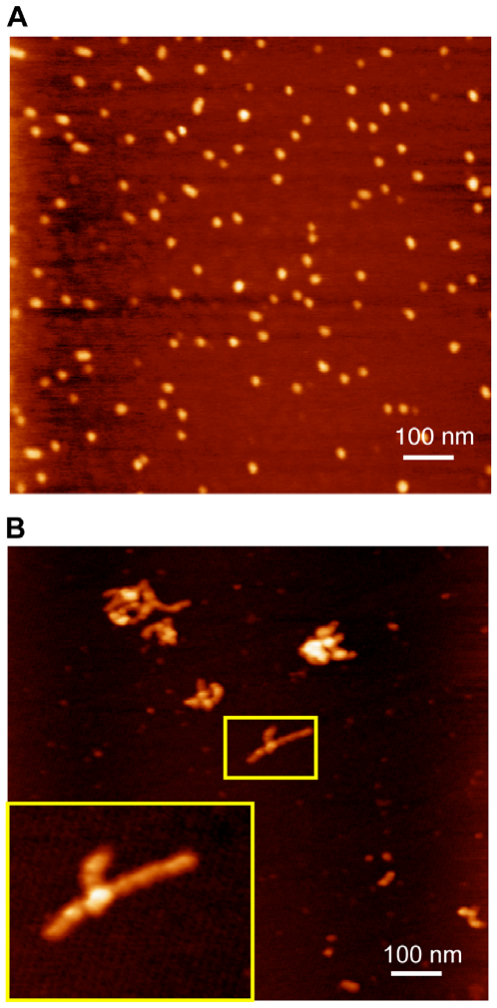
Atomic force microscopy. (A) Visualization of E7SOs. The xy scale corresponds to 1 µm. (B) Visualization of E7C treated with EDTA for 2 hours. The xy scale corresponds to 1 µm. The white square inset indicates the zoom region.

In a previous work [15], we had shown that E7SOs are spherical and homogeneous in size, as judged by electron microscopy. We now analyze the oligomers using tapping mode atomic force microscopy (AFM), and observed a homogenous spherical population with a diameter <19 nm for the full-length protein (Figure 7A). The diameter measured by AFM is smaller than that observed with electron microscopy [15], which can arise from tip deformation, but it is consistent with DLS measurements presented here (16.9±2.2 nm, Figure 2A).

However, images of E7C after 2 hours of incubation with EDTA show that the E7C oligomers have a *worm-like* amyloid structures morphology with a contour-length of 50–150 nm (Figure 7B). These results indicate that removal of zinc from the E7C domain triggers the formation of oligomers but with different characteristics from those of the full-length E7 protein. E7C oligomers have inhomogeneous size and are the precursors of worm-like amyloid structures while E7SOs have a spherical shape, are homogenous in size and remain stable in solution.

Otherwise, the N-terminal domain of E7 has an intrinsically disordered nature, it does not contain any metal, and we previously showed that it is monomeric at 100 µM concentration [19]. In addition, NMR experiments show that E7N is monomeric at 3 mM concentrations (not-shown).

## Discussion

The E7 oncoprotein from human papillomavirus is the major transforming protein of the virus, with counterparts in other DNA tumor viruses. We have been investigating its biochemical properties (structure-function) in connection with its biological role. The protein binds to a large number of cellular targets, and this property lies largely on its structural plasticity arising from the intrinsically disordered nature of its N-terminal domain, and in fact, it turned out to be an excellent model for IDPs [17], [18], [19], [43]. A salient feature was its ability to self-assemble into spherical oligomers upon removal of a tetrahedrically coordinated zinc atom, and we showed that these structures are formed within cell lines and cancerous tissue [27].

The ability to form stable spherical and soluble oligomers with amyloid-like properties, and the well known fact that such type of oligomers are universal intermediates in amyloid pathways [44], establishes it as a model for understanding general amyloid intermediates assemblies. Moreover, spherical oligomers, not insoluble fibers, are believed to be the more toxic species [1], [45], providing more interest to the challenge of dissecting its assembly mechanism.

With this in mind, we made use of a number of different and complementary spectroscopic and biophysical probes to understand the complex polymer chemistry behind this mechanism. The reaction starts from the addition of a metal chelator which ultimately leads to oligomerization. The process is rather slow (no events observed in experimental dead time), and involves changes in secondary, tertiary and quaternary structure, with the presence of a significant lag, followed by an elongation phase, ultimately leading to a homogeneous spherical oligomer (E7SOs), with no parallel soluble or insoluble routes.

For the analysis of the reaction, we used a kinetically controlled model (KL) in order to define the parameters at each stage [28]. E7 is a weak dimer and exists in a fast pre-equilibrium with a monomeric species. This species interacts with the chelator which slowly and gradually reacts with the zinc atom, forming an ensemble/collection of ternary E7-zinc-chelator complexes (“pre-nucleus intermediate”), and replacing each of the thiolate bonds present in the tetrahedrical coordination from the initial native state of the protein. This slow process constitutes the lag phase which terminates by the complete release of the zinc atom and the generation of an apo-monomeric Z-nucleus, which grows gradually by sequential second-order additions of monomers into the final E7SOs.

The evidence presented throughout the work indicates that the Z-nucleus is rather structured, even though the native state of E7 must necessarily change drastically upon the removal of the structural zinc atom. Moreover, even when all zinc-coordinated cysteines are modified by the specific reagent PMPS, the assembly does not take place, strongly suggesting that a partially folded intermediate conformation is required and that the cysteine residues either participate in assembly [46] or the latter is sterically hindered by the presence of the PMPS moiety. In support to this, this species is able to readily recapture free zinc upon removal of the cysteine modifier.

**Figure 8:**
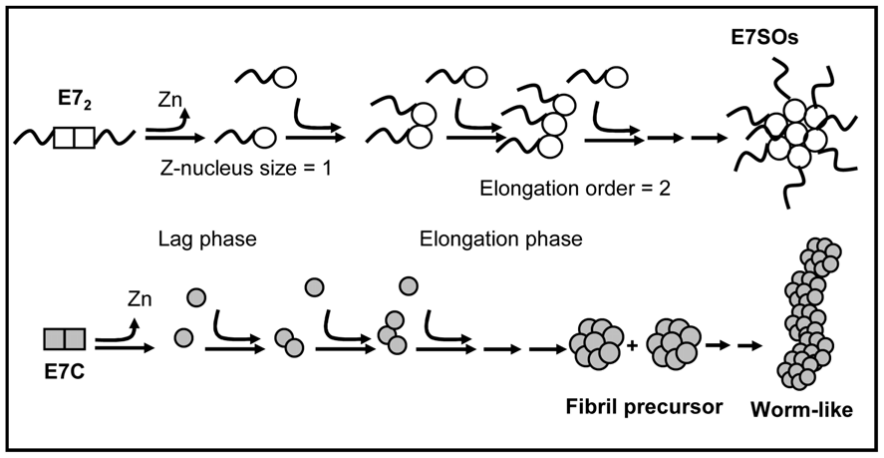
Schematic representation of E7SOs self-assembly route and amyloid route on E7C. The scheme shows the hallmarks features of the E7SOs assembly, including the slow z-nucleus formation phase and the second order polymerization reaction. E7C that contain the assembly domain but is devoid of the IDD N-terminal region is prone to form worm-like structures.

By showing that the zinc-containing globular C-terminal domain alone can self-assemble, we demonstrate that the oligomerization determinant lies within this domain. However, the process is not identical to the full-length protein, as the E7C oligomers are heterogeneous and ultimately lead to insoluble aggregation, by forming worm-like structures, similar to those observed in amyloid routes [47]. Therefore, this modular oncoprotein oligomerizes by its C-terminal domain and requires its N-terminal intrinsically disordered domain to avoid undergoing irreversible aggregation into worm-like amyloid structures. This domain is highly acidic, and bears several interaction sites, including its main target retinoblastoma, as well phosphorylation and potential PEST degradation site, and these remain exposed to the milieu. A picture emerges of a structured core (E7C) surrounded by a dynamic/fluctuating IDD (E7N). In the complex environment of the cell, and given the fact that it can interact with such a large number of targets, we hypothesize that E7 is likely to form hetero-oligomers [27]. Moreover, we had shown that E7SOs display non-specific chaperone holdase activity [16]. Figure 8 integrates the main features of the two oligomerization mechanisms, stressing how the presence of the E7N IDD prevents the progression into an amyloid route.

The present work dissects the self-assembly mechanism of a model viral oncoprotein with IDP properties. Interestingly, the work goes beyond the role of the functional assembly of this oncoprotein and provides insights into different biological and chemical processes. The first series of implications relates to polymer assembly. A fragment as small as 5 kDa undergoes high efficiency and high fidelity ordered self-assembly, triggered by the removal of a structural zinc, which nevertheless does not lead to unfolding or aggregation.

An interesting conclusion is that a similar assembly mechanism holds for viral capsids, thought to be very symmetrical and regular structures [29]. Most importantly, spherical self-assemblies are at the center of amyloid routes, as obliged intermediates (often the toxic species) in the formation of amyloid fibers.

An ordered self-assembly triggered by zinc removal make us wonder how this takes place within the cellular environment. We have defined controlled “artificial” experimental conditions of temperature, pH, protein and chelator concentration, but we can speculate about the “natural” conditions. An obvious one is the concentration, which is affected by the balance between synthesis and degradation, but also influenced by molecular crowding. Since free zinc concentration in cells is minimal, the process could be assisted by a metal removing protein such as metallothioneins or metal chaperones [48]. Given that we have shown that the oligomers exist in their natural host cells in high levels specifically within the cytosol, the self-assembly event would then be modulated by a cellular zinc chelator, providing a regulatory role to this particular zinc, beyond its structural role in stabilizing a particular non-oligomeric fold.

In summary, this modular partly intrinsically disordered viral oncoprotein undergoes an ordered self-assembly mechanism that shares fundamental features with other natural macroassemblies such as viral capsids and amyloid intermediates, can take place and could be modulated within cells, and provide a platform for nanotechnological applications. Some of these include novel scaffolds for therapeutic applications such as self-assembly peptides used in biomaterials for regenerative medicine [49] or slow release of cytokines [50]. In fact, we have recently demonstrated that the E7SOs can be used as therapeutic vaccine candidates for HPV related neoplasic lesions [51].

## Materials and Methods

### Proteins Expression and Purification

Recombinant E7 HPV-16 protein was expressed and purified as previously described [17]. The E7 HPV-16 C-terminal domain, spanning residues 40–98, was expressed and purified as previously described [52].

### Circular Dichroism (CD)

Far-UV CD measurements were carried out on a Jasco J-810 spectropolarimeter using a Peltier temperature-controlled sample holder at 25°C in a 0.1 cm path length cell with a protein concentration of 15 µM. All the measurements were performed at 10 mM sodium phosphate pH 7.0 and 1.0 mM DTT. Assembly kinetics were followed by monitoring at 202 nm and 216 nm.

### Fluorescence Measurement

Fluorescence measurements were performed using a Jasco J spectrofluorimeter (Nikota Japon). In order to compare the changes in the fluorescence intensity during assembly at different protein concentration, the photomultiplier voltage and the emission and excitation band pass were kept constant in the measurement of different protein concentrations samples. All the measurements were performed at 10 mM sodium phosphate pH 7.0 and 1 mM DTT. The ThT assays at different protein concentrations were performed keeping ThT concentration constant at 20 µM. ThT kinetics were followed at 490 nm and 446 nm emission and excitation wavelength respectively. The ANS fluorophore was used at 60 µM, and the kinetic was carried out at 463 nm emission and 370 nm excitation wavelength. Kinetics following Tyr fluorescence were carried out at 305 nm emission and 280 nm excitation wavelength. In all cases, the starting point corresponds to the addition of EDTA or TPEN chelator.

### Nucleus Size Calculation

In the KL model, the relationship between the concentration of the E7SOs and the free protein at a certain time during the assembly is given by the equation 1: [E7SOs] = k*[E7free]^n^. This relationship is valid in the time interval, after the lag phase and before reaching the steady state. In the equation 1, k is a proportionality constant and n reports the nucleus size. The nucleus size *n* is calculated from the slope of a log-log plot of E7SOs and E7 free molar concentration. Each straight line was obtained calculating the E7SOs and E7 free concentrations obtained at a single time for different initial total protein concentrations. E7 free concentration is monomeric concentration. This model was applied in the ThT fluorescence kinetics at different protein concentrations. The ThT fluorescence signal is proportional to the E7SOs assembly: [E7SOs]∼ThT signal. The free protein was taken from: [E7free]∼[E7]initial*(1-ThTsignal) [28].

### Dynamic Light Scattering (DLS)

DLS measurements were carried out on Zetasizer Nano S DLS device from Malvern Instruments (Malvern). Measurements were performed in 10 mM sodium phosphate pH 7.0 and 1.0 mM DTT. E7 and E7C were filtrated with Ultrafree-MC microcentrifuge filters 0.22 µm Millipore before measurements were done. E7 and E7C protein concentration were kept at 15 µM. The temperature was maintained at 25°C by Peltier control system. Results were processed employing the software package included in the equipment. All the points in the kinetics assay were carried out by averaging a set with 6 measurements duration 10 seconds each one and the delay between set was 60 seconds.

### PAR-PMPS Assay

Briefly, 600 µM of the mercurial reagent PMPS was added to 10 µM of E7 dimer, leading to the release of Zn. The secondary structure of PMPS modified apo-E7 was evaluated by far UV CD spectroscopy [53]. After incubated during 1 hour, DLS measurement was performed to analyze the PMPS modified apo-E7 oligomerization state.

To confirm the Zn release from E7 protein upon PMPS addition, we performed the same experiment but in presence of 100 µM PAR. PAR reagent form a complex with Zn that can be quantified spectrophotometrically and the time trace of Zn (PAR)_2_ complex formation was obtained by recording absorbance at 500 nm. When indicated, PMPS and DTT were added to a final concentration of 600 µM and 1.2 mM, respectively. As a control experiment, we confirmed that the DTT concentration used does not interfere with the formation of the PAR-Zn, as is expected (Not shown).

### Atomic Force Microscopy (AFM)

For AFM imaging, E7SOs and E7C were performed at 2 ng/µL and 10 ng/µL respectively, in buffer containing 10 mM HEPES pH****7.0 and 1.0 mM MgCl_2_. Ten microliters of the sample was deposited onto freshly cleaved mica. After 5 minutes the samples was gently washed with 1 mL of Milli-Q water to remove molecules that were not firmly attached to the mica and blow-dried with nitrogen. Tapping-mode AFM was performed using a Nanoscope III Multimode atomic force microscope (Digital Instruments, Veeco Metrology, Santa Barbara, CA) using J-type piezoelectric scanner with maximal lateral range of 120 µm. Microfabricated silicon cantilevers 125 µm in length with a force constant of 40 N/m were used (Nano Devices, Veeco Metrology). The images (512 pixels) were captured with a scan size between 0.5 and 3.0 µm at a scan rate of 1–2 scan lines. Images were processed by flattering using Nanoscope software (Digital Instruments), which was used to remove background noise. WsxM 4.0 beta 2.1 software was used to analyze the images.
